# Ultrafast *in cellulo* photoinduced dynamics processes of the paradigm molecular light switch [Ru(bpy)_2_dppz]^2+^

**DOI:** 10.1038/srep33547

**Published:** 2016-09-20

**Authors:** Alejandro De la Cadena, Dar’ya Davydova, Tatiana Tolstik, Christian Reichardt, Sapna Shukla, Denis Akimov, Rainer Heintzmann, Jürgen Popp, Benjamin Dietzek

**Affiliations:** 1 Leibniz-Institute of Photonic Technology Jena, Albert-Einstein-Straße 9, 07745 Jena, Germany; 2 Institute of Physical Chemistry and Abbe Center of Photonics, Friedrich Schiller University Jena, Helmholtzweg 4, 07743 Jena, Germany; 3 Department of Internal Medicine IV, Division of Gastroenterology, Hepatology and Infectious Diseases, Jena University Hospital, Erlanger Allee 101, 07747 Jena, Germany

## Abstract

An *in cellulo* study of the ultrafast excited state processes in the paradigm molecular light switch [Ru(bpy)_2_dppz]^2+^ by localized pump-probe spectroscopy is reported for the first time. The localization of [Ru(bpy)_2_dppz]^2+^ in HepG2 cells is verified by emission microscopy and the characteristic photoinduced picosecond dynamics of the molecular light switch is observed *in cellulo*. The observation of the typical phosphorescence stemming from a ^3^MLCT state suggests that the [Ru(bpy)_2_dppz]^2+^ complex intercalates with the DNA in the nucleus. The results presented for this benchmark coordination compound reveal the necessity to study the photoinduced processes in coordination compounds for intracellular use, e.g. as sensors or as photodrugs, in the actual biological target environment in order to derive a detailed molecular mechanistic understanding of the excited-state properties of the systems in the actual biological target environment.

A milestone in the search for new alternatives to the current clinical drugs and therapeutic methods used in the treatment of cancer is to reduce systemic cytotoxicity of the applied drugs. A currently discussed route to accomplish this goal is to use photoactivated chemotherapeutic agents (PACT), e.g. photoactive d-block metals^1^,^2^,^3^,^4^, which allow precise temporal and spatial control of the therapeutic action by light irradiation of the diseased tissue. Promising results in this respect have been obtained with Ru(II) polypyridyl complexes bearing strained ligands^1^,^2^,^3^,^4^,^5^,^6^,^7^,^8^,^9^,^10^,^11^,^12^,^13^,^14^. These complexes are stable in the dark and become cytotoxic when exposed to visible light (>450 nm). Upon photo-excitation of the complexes the strained ligands become labile and eventually detach from the photoactive metal ion^4^,^14^. The resultant non-occupied coordination sites of Ru(II) can form adducts with DNA, which in turn becomes structurally distorted, hence, inducing cytotoxicity. Recent progress in the development of Ru(II)-based photodrugs has led to some systems with superior potencies compared to cisplatin^2^,^7^,^8^,^11^,^12^. Structurally modified Ru(II) polypyridine complexes could be effectively employed as prodrugs in hypoxic tissue because there is no need to generate singlet oxygen but cytotoxicity is induced by a light-induced ligand exchange reaction. Also, as diagnostic tools, transition metals (specially Ru(II) due to its well established chemistry) coordinated to polypyridine ligands are candidates for the synthesis of alternative *in cellulo* emission markers. Among their attractive features as cellular probe agents are their large Stokes shift, long phosphorescence life times, low metal to ligand charge transfer (MLCT) excitation energies in the visible region (avoiding the UV light which induces DNA damage)^15^, high quantum yields, photo- and chemical-stability, water solubility, low cytotoxicity and inertness, i.e. low-ligand exchange rates (similar to those of cellular division processes). Furthermore, the cellular localization can be controlled by choosing the ancillary ligands and their substitution pattern^16^,^17^,^18^.

In this respect [Ru(bpy)_2_dppz]^2+^ (**1**) (bpy = 2,2′-bipyridine and dppz = dipyrido [3,2-a:2′,3′-c]- phenazine) can be considered the long-standing paradigm system as it is known as luminescent DNA marker and the dppz-ligand presents a lead structure for the development of DNA-intercalating ligands^19^,^20^,^21^,^22^ (see Fig. 1). The paradigm molecular light switch compound **1** intercalates into adjacent DNA base pairs through its dppz ligand with a binding constant of ca. 10^6^ M^−1 ^21,22,23,24. The intercalation was proven by photophysical and NMR studies^25^,^26^,^27^,^28^ and crystal structures of Ru(II)-dppz-DNA adducts have been reported quite recently^29^. The photophysical properties of the archetypical molecular light switch compound **1** and related complexes have widely been studied in (aqueous) solutions in absence and presence of DNA^23^,^26^,^30^,^31^,^32^,^33^,^34^ and embedded into phospholipid vesicles^35^,^36^. Other studies based on luminescence imaging and flow cytometry have focused on the cellular uptake into cells, their intra cellular localization and their cytotoxicity of Ru(II)-polypyridine complexes^16^,^18^,^37^,^38^,^39^,^40^,^41^. These studies showed that uptake of Ru(II)-polypyridyl complexes is generally enhanced when the structural integrity of the cell gets impaired, however, uptake of compound **1** and related systems into cells and nuclei have been shown^37^,^42^,^43^. The results described in the literature point out that the photophysics (and in some cases the reactivity^41^) of Ru(II)-based complexes highly depends on the environment of the complexes^16^,^18^,^37^,^38^,^39^,^40^,^41^. However, these conclusions are derived from photophysical studies in model solvents, while the ultrafast photophysics of compound **1** - and other Ru(II) based molecular markers or photodrugs for photodynamic therapy or small molecule release - has not been studied *in cellulo* up to now.

To address this issue, the work at hand presents the first study of the ultrafast photophysical properties of compound **1** *in cellulo* using ultrafast transient absorption microscopy^44^,^45^. Thus, this study addresses exemplarily the question whether the photophysics of a Ru(II) polypyridine complex *in cellulo* differs from the photophysics obtained from solution studies. Insofar it presents a corner stone in perspectively studying the function determining photophysical processes in photodriven cis-platin analogs, which have recently been introduced^1^,^3^,^4^,^5^,^6^,^7^,^8^,^9^,^10^,^11^,^12^,^13^,^14^.

## Results and Discussion

**Figure 1:**
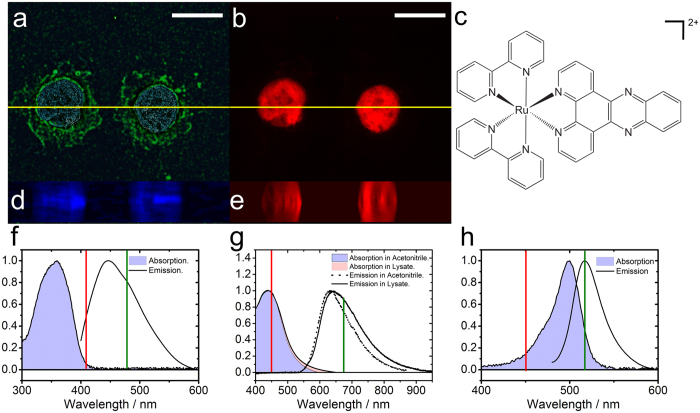
HepG2 cells incubated with DAPI, Alexa and compound 1. (**a**) Emission of DAPI (blue) and Alexa (green) indicate the nucleus and the actin cytoskeleton. (**b**) Emission of compound **1** (red). On the SIM images shown on panels a and b the scale bars represent 10 *μ*m. (**c**) Molecular structure of compound **1**. (**d**,**e**) Are z-stacks of the images taken along the yellow lines shown in panel a,b, respectively. *λ*_*exc*_ = 405 and 450 nm, *λ*_*DAPI*_ = 461 nm, *λ*_*AX*_ = 519 nm and *λ*_*Ru*_ = 670 nm. (**f**–**h**) Show the normalized absorption and emission spectra of DAPI (in water), compound **1** (in a cell lysate of HepG2 cells and in ACN) and Alexa (in water), respectively. Excitation and emission wavelengths are shown in red and green respectively.

As a first step to study the photoinduced dynamics of the paradigm system compound **1** *in cellulo*, the distribution of the Ru(II) dye in the cells is validated. To this end HepG2 cells were co-stained with compound **1** and the cellular markers DAPI (4′, 6-diamidino-2-phenylindole) and Alexa (Alexa Fluor 488 Phalloidin) to visualize the nucleus and the cytoskeleton, respectively. Successive imaging of individual cells at different focal planes revealed the intracellular distribution of compound **1** (see Fig. 1). The data show that compound **1**, a lipophilic cation with a length of ca. 17 Å^46^, is found in the nucleus of the fixed HepG2 cells – a result in line with literature reports by Thomas and coworkers^40^, who report [(bpy)_2_Ru(tpphz)Ru(bpy)_2_]^4+^ (tpphz = tetrapyrridophenazine) to be a valuable dead-cell stain. It should be pointed out that phosphorescence of compound **1** is observed co-localized with the emission of DAPI – not only indicating accumulation of the dye in the nucleus but also corroborates the intracellular light-switch mechanism, i.e. switching on the phosphorescence of the dye by exclusion of water molecules from the pyrazine nitrogens upon interaction with DNA^35^,^40^,^47^.

For fixing the cells, we followed procedures and used reagents which have been proved to yield highest histological quality^48^. The fixation agent employed in this study was a formalin solution (HT50-1-128; Sigma Aldrich) which is the most commonly used fixative agent for optical microscopy. In addition, formaldehyde is considered the gold standard of fixatives for histology and immunohistochemistry due to its outstanding performance to retain cellular constituents on a state that resembles in good approximation the characteristics of living cells^48^,^49^. After the cells have been incubated with compound **1** for 24 hours the medium was removed. The grids that contained the cells were washed twice with PBS buffer. Subsequently the cells were fixed by treating them with a 10% formalin solution for 60 minutes. Finally, the samples were washed with PBS buffer and were dried in sterile conditions.

To study the photoinduced dynamics of compound **1** *in cellulo* without the interference from DAPI or Alexa, HepG2 cells were incubated exclusively with the transition metal complex. Incorporation of the dyes into the cells was – again – confirmed by laser scanning emission microscopy (see Supplementary Fig. 1). In HepG2 reference cells, which were not incubated with the Ru(II) complex, the characteristic phosphorescence at around 670 nm was not observed. However, it should be mentioned that appreciable autofluorescence of some HepG2 cells was recorded under the experimental conditions of this study. This cell-intrinsic signal (see Supplementary Fig. 2)^50^ could be spectrally separated from the emission of compound **1** by using a long pass filter (cut-off *λ* = 650 nm).

**Figure 2:**
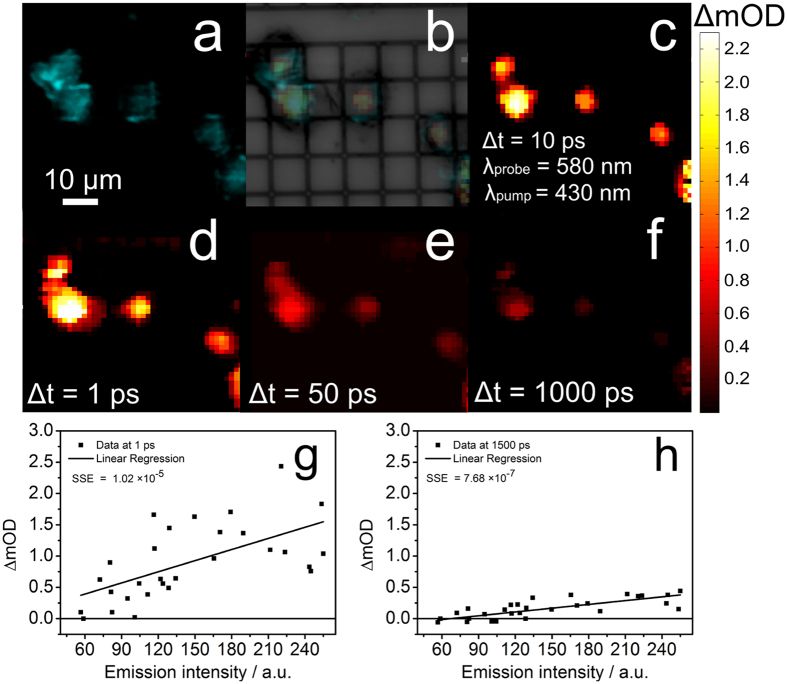
Stained cells imaged by (**a**) Confocal fluorescence microscopy image exciting at 450 nm and monitoring the emission at 670 nm. (**b**) Overlay of panels a,c. (**c**) Transient absorption (*λ*_*pump*_ = 430 nm and *λ*_*probe*_ = 580 nm). (**d**–**f**) Transient absorption images of the identical sample area but at increasing pump-probe delays. (**g**,**h**) Correlation of the transient absorption signal with the emission intensity exemplary shown for two different delay times, i.e. 1 and 1500 ps.

In order to get a first glimpse on the ultrafast excited state processes giving rise to the image contrast in pump-probe microscopy, the signal intensities obtained from transient absorption microscopy and confocal fluorescence microscopy are correlated (see Fig. 2 panels g,h and Supplementary Fig. 3). This analysis reveals an overall correlation between the signal strengths obtained from transient absorption and emission microscopy, i.e. intense transient absorption signals (ΔmOD > 0.3 mOD) are observed in positions of the cells, in which also strong phosphorescence signals of the complex are recorded. This result is expected as both microspectroscopic modalities are related to the presence of compound **1** and scale linearly with the concentration of the samples. Nonetheless, it should be kept in mind that fluorescence microscopy explores only the emissive ^3^MLCT_*phen*_ state of those complexes whose pyrazine nitrogens are protected from water^23^,^30^,^33^, while transient absorption microscopy in principle samples all accessible excited states of all complexes, in particular also those whose long lived emissive state is quenched due to the interaction of the phenazine nitrogens with water^23^. In the latter case the differential absorption signals at long delay times are (close to) zero, but signals at short delay times still contribute to the differential absorption image. To quantitatively correlate the results from transient absorption microscopy and confocal fluorescence microscopy, emission intensities were estimated by calculating the mean of the values from the pixels contained in a single square 5 × 5 matrix for every point of an image, while the transient absorption value for the same point at different time delays was computed. The spatial convolution in correlating the emission intensities with the transient absorption signals was intended to compensate any possible spatial mismatch between the images obtained from the two different microscopes. The resultant data was fitted using a linear regression model. A linear correlation between the emission intensity and the differential absorption signal on fast (delay time < 10 ps) and slow (10 ps < delay time) dynamics scales is observed, however, the goodness of the fit is improved with the analysis done at longer delay time scales. (One order of magnitude better correlations are achieved for the analysis on the pump-probe data when delay times are chosen to be longer than 50 ps as compared to shorter delay times, see Fig. 2 and Supporting Information 2). This finding is due to the fact that the differential absorption signal at long delay times and the complex’s emission probe the identical molecular state, i.e. the ^3^MLCT_*phen*_^23^,^30^,^33^.

**Figure 3:**
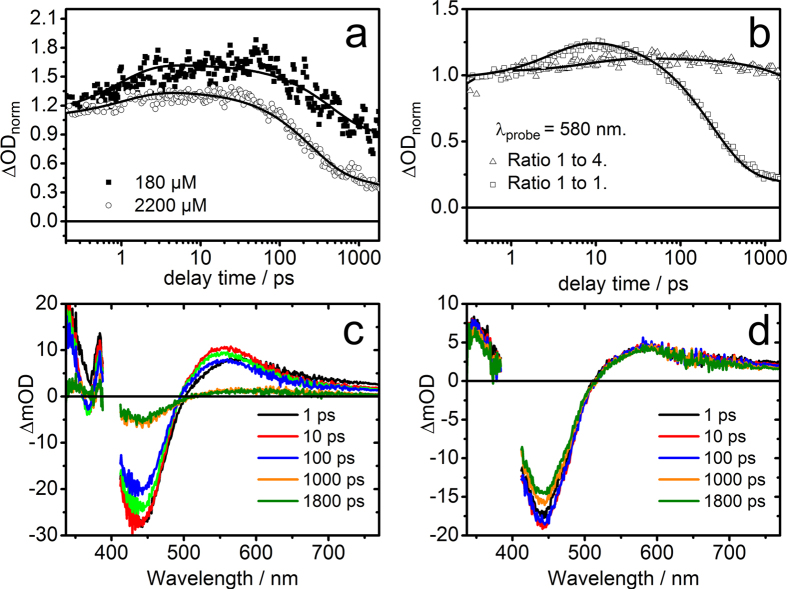
(**a**) Spectrally averaged transient absorption kinetics recorded in the spectral range of 480–670 nm upon excitation at 470 nm at two different concentrations of compound **1** dissolved in lysate solution of HepG2 cells. Note the appearance of a third component (whose ground state recovery is not resolved) starting around 100 ps, in addition to the fast and slow components observed when compound **1** is dissolved in water. (**b**) Comparison of the ultrafast kinetics traces of compound **1** recorded in DNA environments at two different Ru-complexes/Nucleotide ratios (*λ*_*pump*_ = 400 nm and *λ*_*probe*_ = 580 nm). (**c**,**d**) Selected transient absorption spectra of compound **1** in DNA environments at 1:1 and 1:4 Ru-complexes/Nucleotide ratios respectively (*λ*_*pump*_ = 400 nm).

Having correlated the transient absorption signal intensity at different delay times with the emission intensity, we now continue by studying the excited-state kinetics of compound **1**. Initially the kinetics of the complex in a lysate of HepG2 cells will be considered (see Fig. 3 panel a). The kinetics displays a modest signal intensity increase on a sub-10 ps timescale and a decay of the signal on a some-100-ps timescale leading to a long-lived signal offset. Notably upon reducing the concentration of Ru(II) in the lysate, the some-100-ps decay becomes less pronounced and the relative amplitude of the long-lived component rises. In order to understand this result, the kinetics obtained for compound **1** dissolved in water and in acetonitrile (ACN) were used as basis-functions and linearly combined to yield the kinetics observed in the lysate (see Supplementary Fig. 4). Thus, the result can be understood as two different fractions of complexes being present: one fraction interacting with the DNA of the sonoporated cells and a second one being in an aqueous environment. While the first fraction of molecules gives rise to the long-lived state, the second fraction is responsible for the some-100-ps decay of the signal. In the cell lysate there is a given concentration of DNA fragments available for intercalation of the complex. Hence, increasing the concentration of the Ru(II) in the lysate leads (eventually) to a saturation of all available intercalation sites and the relative weight of the long-lived signal contribution to the overall signals diminishes. In principle the reduced long-lived signal could be due to inter-chromophore interactions, which lead to a quenching of the long-lived excited states. However, the parameters of the experiment, i.e. the overall concentration and the pump power used, are far off from values that have been previously reported to lead to such inter-chromophore quenching. Therefore, we consider the contributions from inter-chromophore effects on the concentration dependent transient absorption kinetics of compound **1** in the lysate negligible^36^.

Transient absorption spectra of compound **1** were recorded when interacting with DNA (438545-06-3; Sigma-Aldrich) at Ru-complexes/Nucleotide binding ratios of 1:1 and 1:4. At a 1:4 ratio it is assumed that all complexes are intercalated into DNA, while at higher complex concentrations the available binding sites might become saturated and hence free complexes contribute to the spectroscopic signatures of the sample. This situation is reflected in the time-resolved transient absorption spectra shown in Fig. 3. Panel c displays the data for the 1:1 ratio, which reveals a partial ground state recovery within the first 2 ns after photoexcitation. This spectral evolution can be accounted for by two distinct kinetic components (characterized *τ*_1_ = 4 ps and *τ*_2_ = 250 ps), which is in line with the results published in literature^33^,^36^. However, this situation changes qualitatively when studying the Ru-complexes in the presence of DNA base pairs in a concentration ratio of 1:4 (see Fig. 3 panel d). Here, only marginal ground-state recovery is observed within the experimentally accessible range of delay times. This is due to the fact that interaction of the dppz ligand into DNA prohibits hydrogen-bonding interactions of solvent molecules with the phenazine nitrogens, which is known to quench deactivation pathways and prolong the lifetime of a ^3^MLCT state. The characteristic spectral signatures of the latter state are reflected in the long-lived transient absorption spectra in Fig. 3 panel d^33^,^36^,^51^.

The kinetics, which exemplary summarize the different excited-state behavior of the complexes when dissolved in the presence of varying concentrations of DNA, are shown in Fig. 3 panel b: Upon photoexcitation at 400 nm, the transient absorption signal recorded at 580 nm reflects the overall number of excited states. As can be seen, at a ratio of 1:1 a fast quenching of the excited states is observed which is reminiscent of the data recorded for 2200 *μ*M of compound **1** dissolved in the cell lysate (see Fig. 3 panel a). On the other hand, 180 *μ*M of compound **1** dissolved in the cell lysate shows a much reduced excited state deactivation, which – in the light of the DNA intercalation experiments – can be interpreted as more complexes interacting with the DNA which is present in the lysate, an evocative of the dynamics observed on the Ru-complex/Nucleotide experiments at a ratio of 1:4.

**Figure 4:**
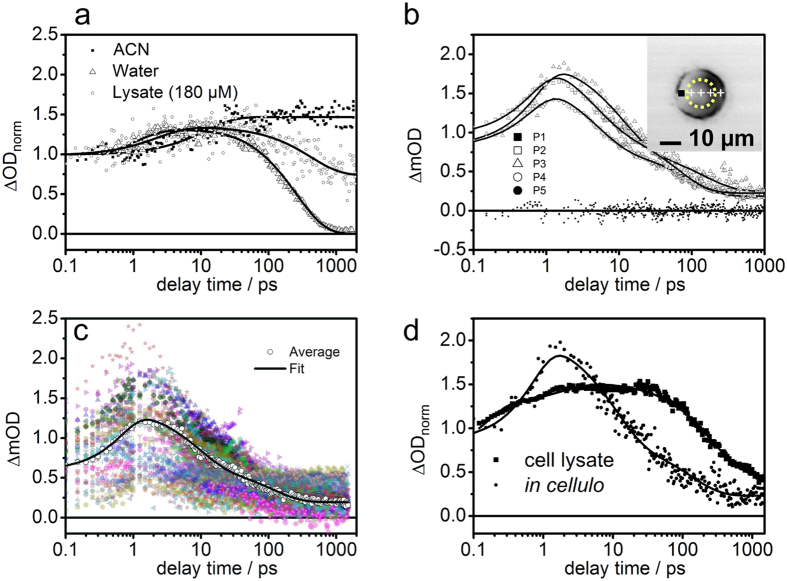
Spectroscopic experiments performed on compound 1 in different environments. (**a**) Integrated transient absorption kinetics recorded in the spectral range of 480–670 nm upon excitation at 470 nm of compound **1** dissolved in lysate solution of HepG2 cells, a protic solvent, water, and an aprotic solvent, acetonitrile. (**b**) Corresponding kinetics traces observed in the indicated positions of the cell displayed on the inset (black square indicates point 1 while the yellow dashed line denotes the nucleus), the data was recorded with *λ*_*pump*_ = 430 nm and *λ*_*probe*_ = 580 nm. (**c**) Ultrafast transient absorption kinetics data set (N = 98) of the experimental results recorded on the nuclei of doped HepG2 cells. (**d**) Transient absorption curves of compound **1** dissolved in lysate solution of HepG2 cells and embedded in a HepG2 cell.

Finally, the excited-state dynamics as reflected in the pump-probe kinetics recorded at different spatial positions within the cell will be considered. In these experiments *λ*_*pump*_ was set to 430 nm to excite a ^1^MLCT transition in the Ru(II) complex, while the probe wavelength was tuned within the range of 550–750 nm. In this spectral range excited-state absorption signals of ^3^MLCT states are probed (given the temporal resolution of our setup), which are characterized by access electron density localized on the dppz ligand^23^,^52^. To obtain sufficient signal-to-noise ratios more than 3 × 10^5^ individual transient absorption measurements were averaged per delay time and in total more than 100 stained cells and control cells were analyzed. Figure 4 exemplary indicates that *in cellulo* pump probe data was recorded in individual HepG2 cells such that voxels inside and outside the nucleus are probed. Exemplary transient absorption kinetics are displayed in Fig. 4 panel b. Notably, in all cells investigated, kinetics with a residual non-zero differential absorption were only observed if the dye in the nucleus was probed. Outside the nucleus typically no transient absorption signal was recorded. Reference experiments included studying the photoinduced dynamics of the Ru(II) complex dissolved in acetonitrile, water and a HepG2 lysate (see Fig. 4 panel a) as well as transient absorption spectroscopy on unstained HepG2 cells (see Supplementary Fig. 5). As can be seen in Supplementary Fig. 5 the control cells show a fast dynamics, however the dynamics does not reveal the long lived photoinduced state observed in doped cells, that would indicate the presence of compound **1**. The central tendency, i.e. the arithmetic mean of the complete data set recorded, of the dynamics exhibited by dyed cells is shown in Fig. 4 panel c while the corresponding data for control cells is presented in Supplementary Fig. 5. In the case of cells incubated with the Ru-dye, the model that accounts for the data includes two kinetic components. One is characterized with a rate constant k_1_ = 0.3 ps^−1^ (3.3 ps), while the second process is described by a rate constant k_2_ = 0.015 ps^−1^ (66.6 ps). The second process (k_2_) results in the formation of long-lived species, whose lifetime is beyond the range of delay times (i.e. 2 ns) accessible in the experimental setup. As indicated by the excellent correlation of phosphorescence emission signal intensity and pump-probe signal recorded at long delay times, the long-lived species is associated with the emissive ^3^MLCT_*phen*_ state characteristic for compound **1** in aprotic solvents or when intercalated into DNA^33^. Interestingly, the dynamics of compound **1** observed *in cellulo* when incorporated into the nucleus resembles the photoinduced dynamics of the complex neither in acetonitrile nor in aqueous solution. It rather resembles a combination of key features observed in either of the other cases (see Fig. 4 panels a–c): The photoinduced dynamics recorded in the nucleus feature a build-up of the transient absorption signal during the first 2 ps after photoexcitation (k_1_) and subsequent decay on a sub-100-ps timescale (k_2_). In particular the latter decay is a characteristic feature of the photoinduced dynamics of compound **1** in a protic solvent. However, at long delay times the transient absorption signal becomes constant within the experimentally accessible range of delay times (i.e., up to 1.5 ns). This feature is assigned to the presence of a long-lived excited ^3^MLCT state in compound **1**, which is also responsible for the observed emission of the complex, when dissolved in aprotic environments^23^,^52^ (Fig. 4 panels a,c).

This kinetic behavior could in principle stem from two distinct sets of molecules within the laser focal volume (ca. 150 attoliters). As the nucleus itself is compartmentalized, only a fraction of the compound **1** that entered the nuclei might intercalate into DNA, thus giving rise to the formation of long-lived (and emissive) ^3^MLCT states, while a second fraction remained in the nucleoplasm. In this environment the complexes might not luminesce but their initial excited state dynamics contributes to the sub-ns kinetics observed. However, using the analogous procedure (as described above) to represent the in-cellulo kinetics by a linear combination of the kinetics recorded in the lysate and obtained for the unstained reference complexes fails, meaning that the resultant linear combination is unable to account for the observed *in cellulo* kinetics. A direct comparison of the transient absorption kinetics of compound **1** incorporated into the nuclei of HepG2 cells with the kinetics observed in the aprotic reference solvent acetonitrile and in the cell lysate partially reveals that the initial rise of the signal contributes with a higher signal amplitude to the overall signal. This indicates that the actual packing of the complex in the biological target environment impacts the excited state dynamics to an extent that cannot be mimicked by model solvents. The impossibility to observe characteristic signals indicative of compound **1** in a protic environment when scanning the cell outside the nuclei, suggests that a considerably higher number of compound **1** molecules are concentrated into the nuclei than in the cytosol. The latter finding is ascribed to the fact that fixed cells were used in this study^39^, assuming that upon fixation the nuclear integrity is impaired leading to an increased uptake of the metal complex. However is worth mentioning that upon transfer of HepG2 cells to the reference quartz grids, the cells were cultivated for 24 hours alongside a solution of compound **1**. After this process, but immediately before fixation, the grids containing the cells were washed with phosphate buffered saline (PBS) with the purpose to remove any detached dead cells and remaining media. Therefore, the doped cells used in this study up-taked the drug while they were alive (however not necessarily viable). This suggests that, for this cell line and under the presented experimental conditions, compound **1** was absorbed by live cells and localized into nuclear compartments.

The ultrafast photoinduced processes in the compound **1**, which can be considered as the paradigm system for Ru(II)-based photodrugs and DNA sensors, has been studied for the first time *in cellulo* by means of localized pump-probe spectroscopy. In summary, the results show that the *in cellulo* ultrafast photoinduced dynamics of compound **1** molecules varied with respect to the cellular location. Thus these results reveal the necessity to study the photoinduced processes in coordination compound based photodrugs in the actual target environment of the drug in order to derive a detailed mechanistic understanding of the interplay between light-induced excited-state physics and physiological action of the photodrug. While this benchmark study was concerned with fixed HepG2 cells current work in progress aims at generalizing these results to living cells and different cell lines.

## Methods

### Pump-probe spectroscopy

Pump-probe experiments on reference samples, i.e. compound **1** dissolved in protic and non-protic solvents as well as in cell lysates, were performed based on the setup described in ref. 53. In these experiments a TOPAS-C (LightConversion) was used to spectrally center the pump-pulses at 470 nm. A white light super continuum in the range between 500 and 710 nm was used as probe, which was generated by focusing a minor fraction of the fundamental laser output into sapphire plate. The polarization between pump and probe pulses was set to magic angle and the differential absorption signals (ΔA) were calculated from subsequent probe pulses measured in pump-on and pump-off conditions. All ultrafast spectroscopic experiments reported here were performed at 26.0 ± 0.5 °C. To ensure sample integrity, steady state absorption spectra were recorded on a Jasco V-670 spectrophotometer after each experimental run. Additionally, for the compound **1**/DNA experiments a setup based on an amplified Ti-Sapphire laser system delivering a train of 100-fs pulses centered at 800 nm with a pulse-to-pulse repetition rate of 1 kHz was employed. Part of these fundamental pulses is used to produce a white light supercontinuum from a calcium fluoride (CaF_2_) plate producing radiation with a spectral range in between 350–850 nm. In the experiments presented above the remaining fraction of the fundamental pulses was used to generate the pump pulses at *λ*_*pump*_ = 400 nm by second harmonic generation in a BBO crystal. Again, the polarization between pump and probe pulses was set to magic angle.

### Pump-probe imaging, localized spectroscopy and emission imaging

In order to investigate the ultrafast dynamics of compound **1** *in cellulo*, laser scanning microscopy and pump-probe spectroscopy were combined in a transient absorption microscope^45^. The technological specifications of this setup can be found in ref. 44 and is schematically shown in Supplementary Fig. 6. In the transient-absorption microscope the samples are scanned in a raster pattern by means of a galvanometer-based scanner system (Cambridge Technologies) that together with the focusing objective (Nikon, CFI Plan Apo Lambda 20X, NA = 0.75, WD = 1 mm) provide diffraction limited optical resolution of the microscope. With this system it is possible to obtain linear absorption as well as transient absorption images and information on the local excited state dynamics by recording pump-probe kinetics in a fixed position of the sample with a temporal resolution of about 1 ps. The samples can be moved along the perpendicular plane of the laser beams with 10 nm precision using a scanning stage (SCAN 75 × 50, Märzhäuser).

Additionally the cellular uptake and cellular distribution of compound **1** in the cells was assessed by confocal laser scanning (using a Carl Zeiss, LSM 510 Meta) and structured illumination microscopy (using a Carl Zeiss, ELYRA-S.1).

### Sample Preparation

As an *in cellulo* model system for the investigation of photoinduced dynamics, the HepG2 human liver cancer cell line was chosen. The cells were cultivated in RPMI 1640 liquid medium with 20 mM HEPES, stable glutamine (FG 1235, Biochrom AG, Germany), 10% fetal bovine serum (10099-133, Life Technologies, Germany) together with 100 units/mL of penicillin and 100 *μ*g/mL of streptomycin (15140, Gibco^®^, Life Technologies GmbH, Germany). The 75 cm^2^ cell culture flasks (658170; Greiner Bio-One GmbH, Germany) were used for cultivation. Cells were maintained in 5% CO_2_ incubator at 37 °C and every two days the medium was changed until approximately 90% confluence was reached. After cells were detached by a 0.05% of trypsin–EDTA solution (L2143; Biochrom AG, Germany), they were transferred onto quartz slides equipped with reference grids and cultivated for 24 hours with 50 *μ*M compound **1** solution or without Ru-complex for control experiments. Finally, fixation of the cells with 10% neutral buffered formalin solution (HT501128; Sigma-Aldrich, USA) for 60 minutes was performed with following washing in distillate water step. Finally, the slides were dried and stored at +4 °C until the microscopy experiments were performed.

For the experiments with fluorescent markers, i.e. DAPI and Alexa Fluor, cells were cultivated onto cover-glasses of 12 mm width (VWR International, US) and incubated with compound **1**(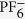)_2_ for 24 hours except for the control cells. The protocol of fluorescent staining includes short washing steps with PBS buffer, PBS buffer with 2% of formalin and PBS buffer again. In a next step the cells were incubated with Alexa Fluor at room temperature for 20 minutes followed by a subsequent bath with PBS buffer. Afterwards, cells were stained with DAPI for 2 minutes at 37 °C and washed twice with PBS buffer. As the final step the cover slips were embedded in a medium of a refractive index similar to the immersion oil (1.518), placed on the glass-slides upside down and glued together on the sides to prevent drying of the samples.

The [Ru(bpy)_2_dppz]^2+^ complexes employed in this study were synthesized according to well established literature protocols^23^,^54^.

## Additional Information

**How to cite this article**: De la Cadena, A. *et al*. Ultrafast *in cellulo* photoinduced dynamics processes of the paradigm molecular light switch [Ru(bpy)_2_dppz]^2+^. *Sci. Rep.* **6**, 33547; doi: 10.1038/srep33547 (2016).

## Supplementary Material

Supplementary Information
