# Supplementary material for: Ultrafast in cellulo photoinduced dynamics processes of the paradigm molecular light switch [Ru(bpy)2dppz]2+
Source: Sci Rep. 2016 Sep 20;6:33547. doi: 10.1038/srep33547 (PMC5028833; doi:10.1038/srep33547)
Supplement: Supplementary Information [file srep33547-s1.pdf]

Ultrafast *in cellulo* photoinduced dynamics processes of the paradigm molecular light switch $[Ru(bpy)_2dppz]^{2+}$ (Supplementary information)

Alejandro De la Cadena[1,2], Dar'ya Davydova[1,2], Tatiana Tolstik[1,3], Christian Reichardt[1,2], Sapna Shukla[1,2], Denis Akimov[1], Rainer Heintzmann[1,2], Jürgen Popp[1,2], and Benjamin Dietzek[1,2,*]

[1]Leibniz-Institute of Photonic Technology Jena, Albert-Einstein-Straße 9, 07745 Jena, Germany.
[2]Institute of Physical Chemistry and Abbe Center of Photonics, Friedrich Schiller University Jena, Helmholtzweg 4, 07743 Jena, Germany.
[3]Department of Internal Medicine IV, Division of Gastroenterology, Hepatology and Infectious Diseases, Jena University Hospital, Erlanger Allee 101, 07747 Jena, Germany.

[*] benjamin.dietzek@leibniz-ipht.de (Benjamin Dietzek)

ABSTRACT

An *in cellulo* study of the ultrafast excited state processes in the paradigm molecular light switch $[Ru(bpy)_2dppz]^{2+}$ by localized pump-probe spectroscopy is reported for the first time. The localization of $[Ru(bpy)_2dppz]^{2+}$ in HepG2 cells is verified by emission microscopy and the characteristic photoinduced picosecond dynamics of the molecular light switch is observed *in cellulo*. The observation of the typical luminescence stemming from a ^{3}MLCT state suggests that the $[Ru(bpy)_2dppz]^{2+}$ complex intercalates with the DNA in the nucleus. The results presented for this benchmark coordination compound reveal the necessity to study the photoinduced processes in coordination compounds for in-cellular use, e.g. as sensors or as photodrugs, in the actual biological target environment in order to derive a detailed molecular mechanistic understanding of the excited-state properties of the systems in the actual biological target environment.

Supplementary information

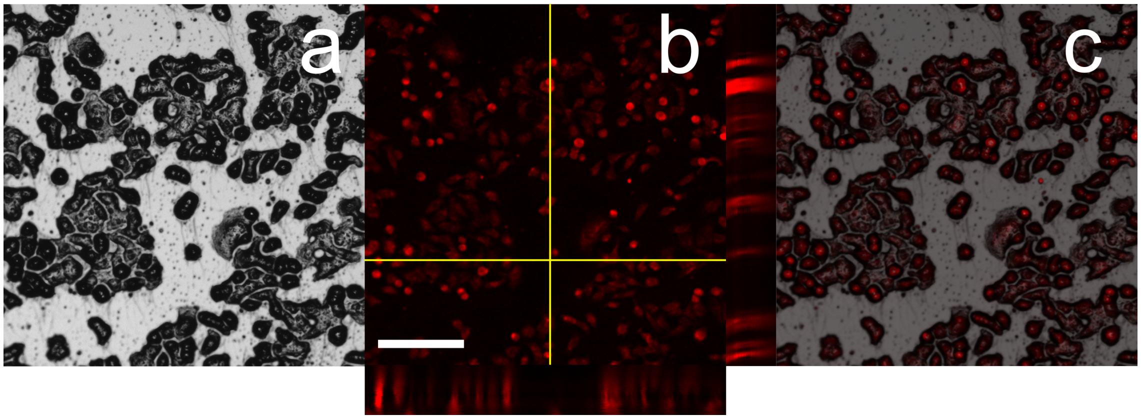

Figure S1. Luminescence imaging of HepG2 cells doped with compound **1**. a) Linear absorption image. b) Emission Image recorded in the spectral range between 650 and 700 nm upon excitation at 453 nm. c) Overlay of the images presented in panels a and b. λ_{exc} = 453 nm. Scale bars represent 100 μm.

To corroborate an efficient uptake and determine the localization of compound **1** molecules in the cells used in this study, HepG2 cell lines were incubated in the absence of any other drug to further record steady state emission images. For this purpose an excitation wavelength of 453 nm was employed to optically set the complexes into a metal to ligand charge-transfer state (MLCT) together with a high-pass (optical) filter whose cut-off wavelength was 650 nm, intended to remove the auto fluorescence of cells and allow only the emission coming from compound **1** relaxing to the ground state by a radiative transition, i.e. the phosphorescence from intercalated compound **1** molecules. The acquired images from these samples exhibited a qualitatively intense and nuclei-localize emission, see Supplementary Figure 1 panel a. Opposite to the notion that compound **1** could remain in the cellular membranes since it is a very lipophilic cation with a considerable length (ca. 17 Å[1]), the results show that the paradigm molecular light switch translocates into the cellular nuclei, as seen in the (oversampled, Δz = 50 nm) z-stack images.

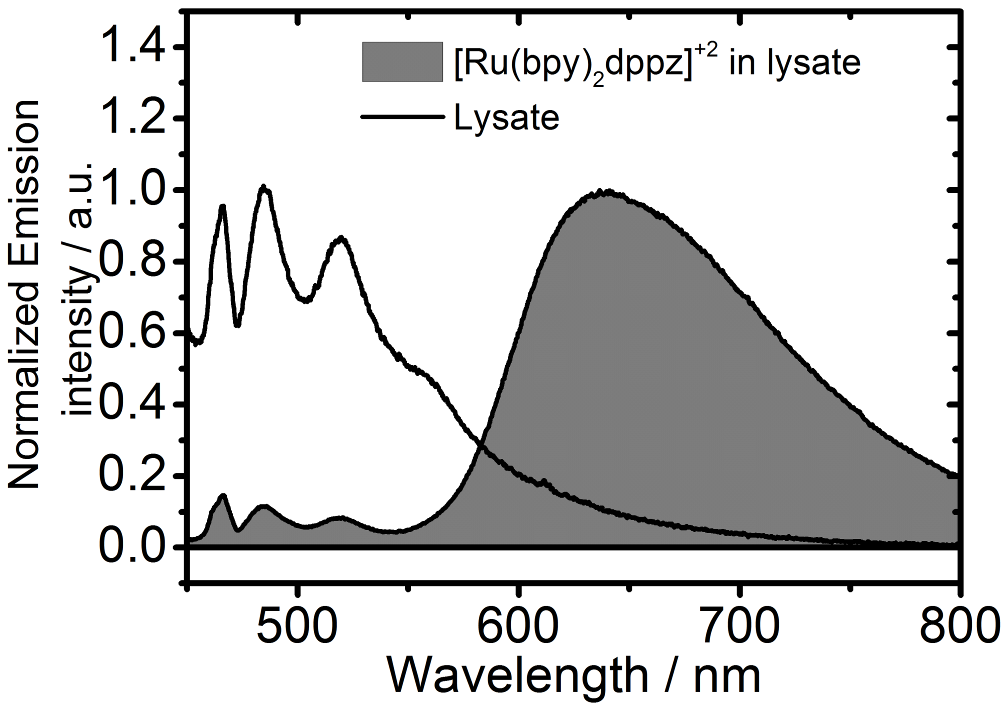

Figure S2. Emission of HepG2 lysate (solid line) and the corresponding signal for a HepG2 lysate containing compound **1** (dark gray surface). λ_{exc} = 400 nm.

The auto fluorescence of cells was observed *in cellulo*. To identify and appropriately remove any interference induced by this signal to the emission of compound **1** in cells, the steady state emission of two HepG2 lysates, one containing 93 μM of compound **1** and the other without complexes, were acquired. For the cell lysate free of Ru-complexes, the maximum emission peak was observed around 490 nm. The same emission peak was observed in the cell lysate containing Ru-complexes in addition to a maximum peak around 650 nm, the latter corresponds to compound **1**. See Supplementary Figure 2.

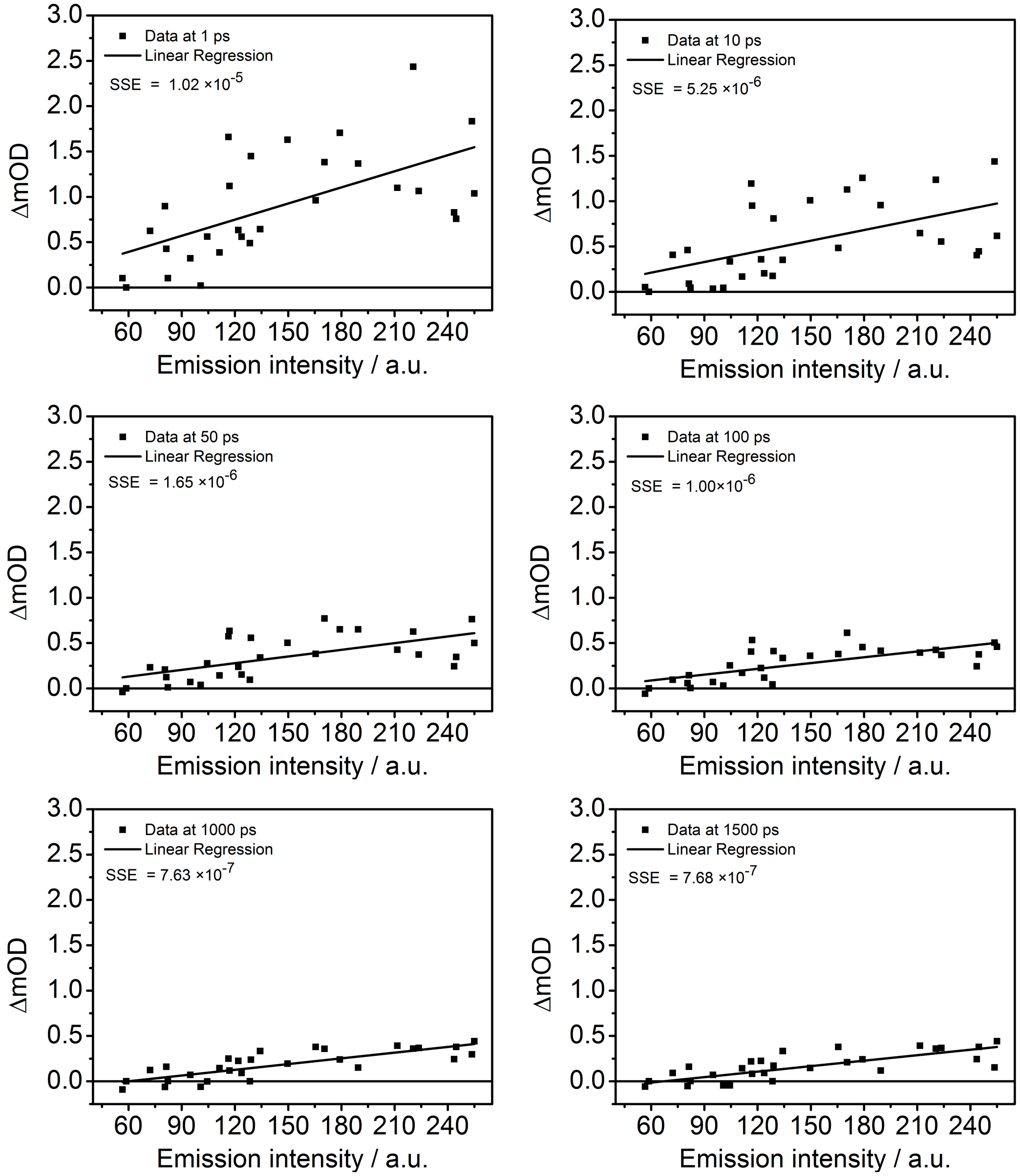

Figure S3. Transient absorption signal recorded at 580 nm upon pumping at 435 nm as function of emission intensities (recorded at 670 nm upon excitation at 450 nm) at different delay times of compound **1** embedded in HepG2 cells.

The quantitative correlation of the data obtained with transient absorption microscopy and emission microscopy is presented. For this purpose the linear dependence of transient absorption signal as a function of the emission intensities was studied by recording the two mentioned spectroscopic signals on different cells at different cellular positions varying solely the delay time of the pump-probe beams. The linear dependence of the data improves at longer delay times. The last is a result of an exploration of the same state ($^3MLCT_{phen}$) by the two different spectroscopic techniques. See Supplementary Figure 3.

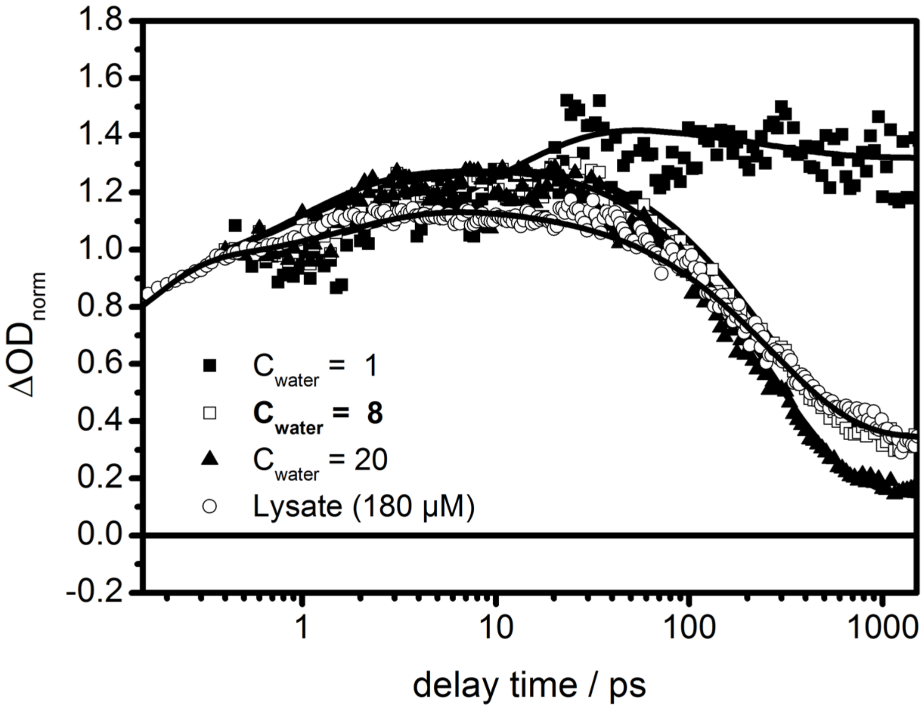

Figure S4. Linear combination of the kinetics traces of compound **1** dissolved in water and in ACN. Note how the linear combination resembles the trend of the kinetic component whose proportionality constant is higher. In this chart C_{water} was varied while C_{ACN} was kept at a constant value of 1.

The kinetics traces of compound **1** dissolved in water and in ACN were linearly combined varying solely a proportionality constants, i.e. C_{ACN} or C_{water} for acetonitrile and water respectively, and in this way give weight to the kinetics components that form the linear combination, see equation S1. As expected, the resulting linear combination resembles the kinetics of that component whose proportionality constant dominates, see Figure 4 panel a and supplementary Figure 4. However the linear combination obtained when C_{water}, eight times larger than C_{ACN}, have a similar trend to the kinetics of compound **1** dissolved in the cell lysate solution.

$$F(t, C_{water}, C_{ACN}) = \left(\frac{1}{C_{water} + C_{ACN}}\right)(C_{water} H_2O_{Kinetics} + C_{ACN} ACN_{Kinetics}) \quad (1)$$

where $H_2O_{Kinetics}$ and $ACN_{Kinetics}$ are the normalized kinetics traces of compound **1** dissolved in water and in ACN respectively.

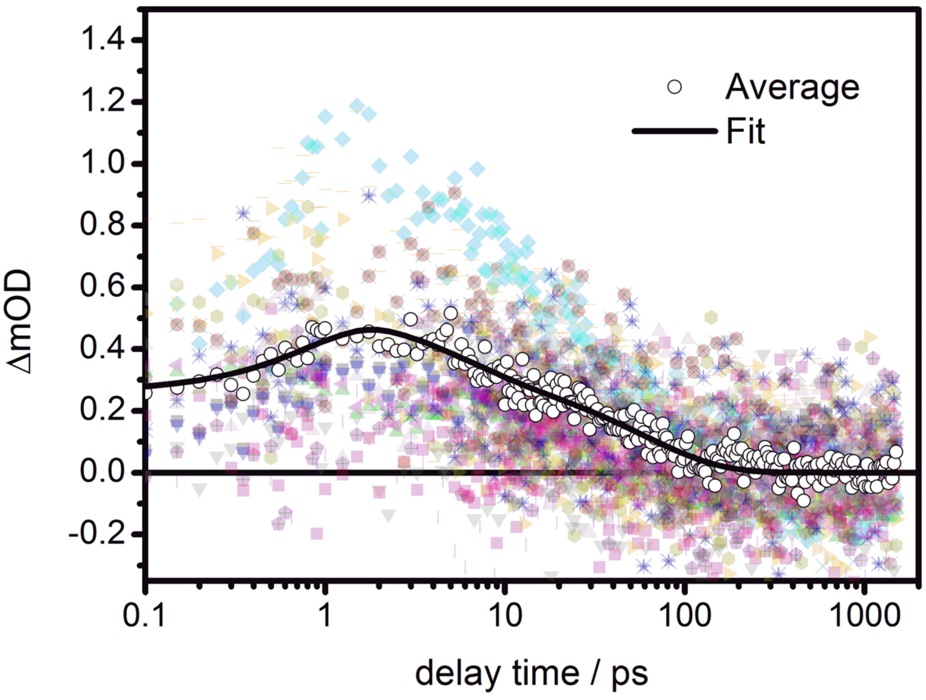

Figure S5. Ultrafast transient absorption kinetics on control cells. Experimental results of the data performed in the nuclei of control HepG2 cells. λ_{pump} = 430 nm and probing in the spectral span of 550 - 750 nm.

The ultrafast transient absorption kinetics on the nuclei of control and doped HepG2 cells with compound **1** are shown in Supplementary Figure 5 and in Figure 4 panel c respectively. The black circles represent the arithmetic mean of the complete data sets (N=72 for control cells and N = 98 for doped cells) while the solid black lines represent the fits of the mentioned means. The data was fitted through two different second order analysis routines,[2] where in the case of doped cells the model computed to fit the data assumes the formation of a species B from a photoexcited species A, whose associate rate constant corresponds to $k_1 = 0.3$ ps^{-1} ($\tau_1 = 3.5$ ps), this second species B decays, with a rate constant k_2 = 0.015 ps^{-1} (66.6 ps), to form a third species whose rate constant is unresolvable with this system. This third species is assigned to the emissive ^{3}MLCT$_{phen}$ state of compound **1**. The kinetics observed in control cells were fitted in a different and simpler way: a photoexcited species A decays directly to the ground state with a $k_{ctrl} = 0.02$ ps^{-1} ($\tau_{ctrl} = 56$ ps).

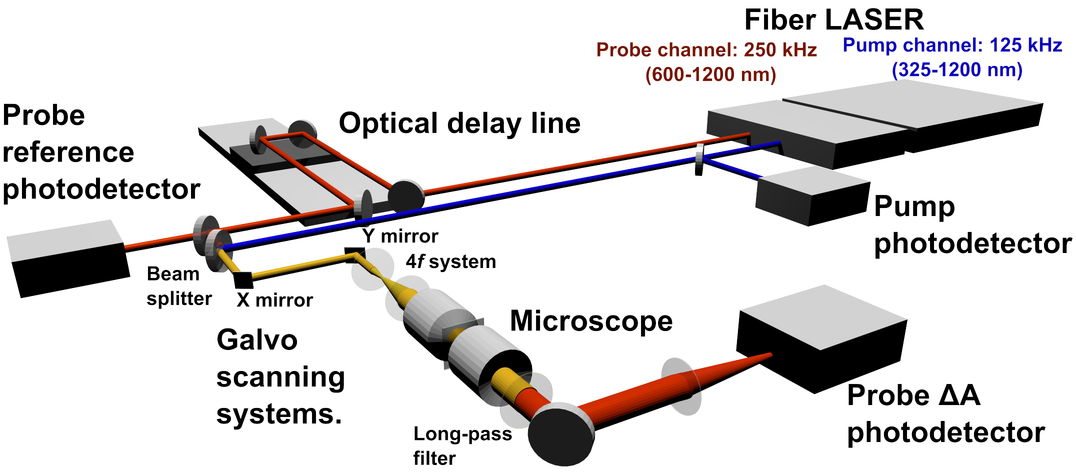

Figure S6. Schematic representation of the ultrafast transient-absorption microscope.

In the transient absorption microscope used in this work, which is schematically represented on Supplementary Figure 6, the laser source consists of two independent (but synchronized) OPAs pumped by an ultrafast high-power fiber-based laser centered at λ = 1030 nm, allowing the OPAs to provide pulses with a time duration of ca. 350 fs and energies around 125 μJ. One pulsed train has a repetition rate of 250 kHz with a tunable spectral-range within λ=550-1200 nm while the other pulsed train oscillates at a frequency of 125 kHz with a tunable spectral-range of λ=325-1200 nm. To calculate the (ΔA) signal with the pump on and pump off condition the 250 kHz channel is employed as probe beam while the 125 kHz channel is used as pump beam. The time delay between the pump and the probe beam is controlled through an optical delay line (PI) and the temporal resolution achieved with this system is ca. 1 ps. The samples are scanned in a raster pattern by means of a galvanometer-based scanner system (Cambridge Technologies) that together with the focusing objective (Nikon, CFI Plan Apo Lambda 20X, NA=0.75, wd = 1 mm) provide diffraction limited resolution. With this system is possible to obtain linear absorption images, localized pump-probe spectroscopy and transient absorption images at a fixed delay time. The samples can be moved along the perpendicular plane of the laser beams with nano-metric resolution, i.e. 10 nm, with a scanning stage (SCAN 75 x 50, Märhäuser).

References

1. Liu, Jin-gang Zhang, Qian-Ling Zhang, X.-F. S. & Ji., L.-N. Interaction of [Ru (dmb)2(dppz)]2+ with DNA: Effects of the Ancillary Ligands on the DNA-Binding. *Inorg. Chem.* **2**, 5045–5050 (2001).
2. Puxty, G., Maeder, M. & Hungerbühler, K. Tutorial on the fitting of kinetics models to multivariate spectroscopic measurements with non-linear least-squares regression. *Chemometrics and Intelligent Laboratory Systems* **81**, 149–164 (2006).
